# A portable negative pressure unit reduces bone cement fumes in a simulated operating room

**DOI:** 10.1038/s41598-022-16227-x

**Published:** 2022-07-13

**Authors:** Gregory T. Carroll, David L. Kirschman

**Affiliations:** Scientific Affairs, Aerobiotix, Inc., 444 Alexandersville Rd., Miamisburg, OH 45342 USA

**Keywords:** Orthopaedics, Polymer chemistry, Health occupations

## Abstract

In this report, we demonstrate a versatile method for the removal of bone cement fumes from the vicinity of health care workers in a simulated operating room. The mixing of two component bone cement in the perimeter of an operating room releases volatile organic compounds (VOCs). The use of localized negative pressure within proximity of the mixing vessel is expected to reduce the concentration of VOCs dispersed near the airway of operating room personnel. A standard two component bone cement formulation was mixed in the perimeter of a simulated operating room. A median VOC concentration of 19 ppm was detected with a portable VOC detector. When a portable negative pressure unit was stationed near the mixing area at distances of 8 and 36 cm from the mixing vessel, the median VOC rise was reduced by approximately 97% and 83%, respectively, relative to the control. The use of a portable negative pressure unit provides a potential increase in the safety for all staff when working with materials that give off VOCs in the operating room.

## Introduction

**Figure 1:**
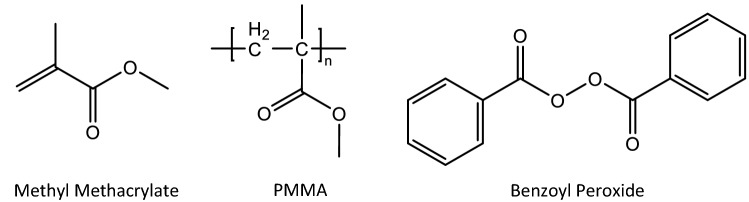
Molecular structures of main chemicals in bone cement.

Bone cement^1–3^ is a synthetic two-component formulation that cures at room temperature upon mixing to form a rigid material that fixates implants. While currently supplied formulations may vary, bone cement generally consists primarily of poly (methyl methacrylate) (PMMA), methyl methacrylate monomer and a polymerization initiator (benzoyl peroxide) (see Fig. 1). For enhanced curing and stability, the formulation will also contain an accelerator (typically a tertiary aromatic amine) and an inhibitor (e.g. hydroquinone). Additionally, antibiotics (e.g. gentamycin), radio-opacifier (typically barium sulfate or zirconium dioxide) and a coloring agent (e.g. chlorophyll VIII) for enhanced visualization are typically present. The polymer-containing component of the pre-mixed system is powder, and the monomer-containing component is liquid. Upon mixing the two components, the methyl methacrylate will gradually undergo an exothermic free radical polymerization, resulting in a high viscosity paste and ultimately a fully cured and rigid material.

Methyl methacrylate, the principle volatile substance in bone cement, is a low molecular weight organic molecule that has a strong odor. It has a boiling point of approximately 101 °C and a vapor pressure of approximately 4 kPa at 20 °C, making it a relatively volatile organic compound. During mixing, some of the methyl methacrylate (and potentially other small molecules present as impurities, additives or side-products and low molecular weight oligomers generated during radical polymerization) evaporates into the air. Heat is generated both as a byproduct of the exothermic polymerization reaction and due to friction caused by mixing. As the viscosity increases, localized heat induced by mixing increases. As the temperature increases, the methyl methacrylate more easily escapes into the air. Throughout the mixing process the odor increases in intensity as the fumes pollute the immediate environment. Note that mixing is performed in the perimeter of the operating room (OR) where downward airflow is not present, leaving health-care workers susceptible to potentially high concentrations of VOCs.

Inhalation of methyl methacrylate may cause respiratory irritation, headache, nausea, vomiting, dizziness and tiredness^4^. Asthma associated with methyl methacrylate has been reported^5–7^. Methyl methacrylate may contain trace impurities of methacrylic acid, which can cause burns to the respiratory tract and pulmonary edema when high concentrations are inhaled. Methyl methacrylate is rapidly metabolized to methacrylic acid in humans. In medical applications, while time-weighted averages are typically less than 100 ppm, maximum exposure levels of up to 374 ppm have been recorded^8^. Methyl methacrylate is considered unclassifiable in regards to carcinogenicity^9^. In general, minimizing chemical exposure regardless of known risks is recommended. The health risks from any given chemical can be upgraded as more data become available. Deploying effective measures to mitigate exposure enhances the working environment in terms of health, safety and productive working.

Various methods for protecting healthcare workers from bone cement fumes have been described. The use of activated carbon impregnated face masks to limit exposure to methyl methacrylate vapors has been reported^10^. Placing small suction tubes near the mixing bowl was described as qualitatively improving inhaled air in the vicinity of the mixing station^11^. Vacuum bone cement mixing has been shown to reduce bone cement fumes in the breathing zone with performance varying depending on the mixing system utilized^12–14^. Pre-packed bone cement mixing systems are a further improvement; however, methyl methacrylate fumes can still be detected when using these systems^15^.

Developing engineering controls to combat exposure to bone cement fumes will improve the occupational environment in ORs. Additionally, such technology would potentially have applications in industrial environments where curing processes^16^ generate VOCs. In general, there is a large interest in reducing volatile and semi-volatile organic compounds in indoor environments due to health and comfort concerns. In hospitals, air cleaning technologies are used to minimize exposure to harmful materials. Portable filtration units^17–19^ which control localized air flow have been deployed in several health-care scenarios for improving air quality. For example, portable air handling units have previously been shown to be effective at removing synthetic aerosols in the size-range of Mycobacterium tuberculosis^20^. Additionally, air decontamination devices that generate negative pressure at point sources have proven effective at removing electrocautery smoke generated during electrosurgery procedures^21^. Portable devices that allow for localized negative pressure offer a potentially flexible and efficient approach to remove bone cement fumes in ORs and other environments.

In this report, we show that bone cement fumes can be detected with a VOC sensor during the manual mixing of a two-component bone cement system and that a portable negative pressure (PNP) unit reduces the concentration of fumes in the localized mixing area. Advantages of the method include the portability of the device and relative ease of incorporating negative pressure into the mixing process in a non-disruptive manner. Additionally, the device can be used for other purposes in the OR including removal of bioaerosols emitted during aerosol generating procedures^22^ and removal of surgical smoke generated during electrosurgery procedures. Although we focus on bone cement in this report, in principle the PNP unit could be used in any environment in which volatile, semi-volatile and particulate matter are generated.

## Materials and methods

**Figure 2:**
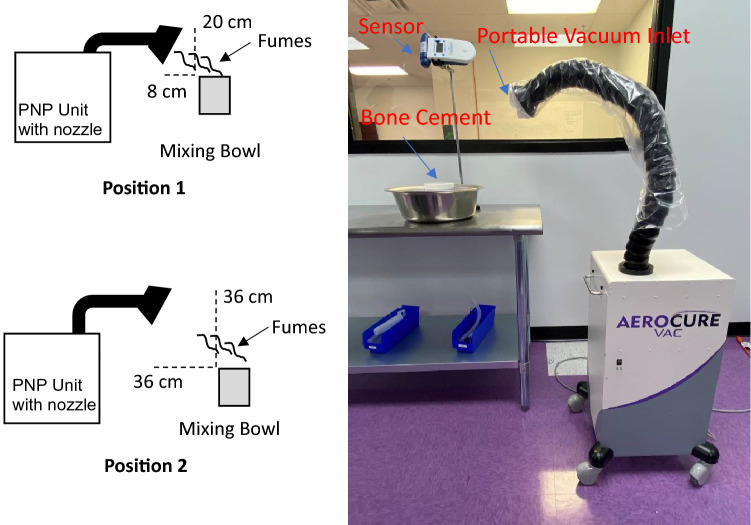
The experimental set-up contains a PNP unit (positioned at two distances), VOC sensor and bone cement.

A two-component bone cement kit (Heraeus) was used. The formulation contained a solid powder component consisting of poly (methyl acrylate, methyl methacrylate) (50.3 g), benzoyl peroxide (0.67 g, 0.0028 mol) and zirconium dioxide (9.0 g, 0.073 mol), and a liquid component (30 ml) containing methyl methacrylate (27.6 g, 0.28 mol) and *N,N*-dimethyl-*p*-toluidine (0.6 g, 0.0044 mol). The components were removed from their enclosures and manually mixed with a wooden spatula in a mixing bowl. Testing was performed in a 50 m^2^ simulated operating room, which included central ceiling-mounted ducts directing HEPA-filtered supply air and four lower wall mounted air return ducts. The system supplied 20 air exchanges per hour (ACH) and 0.03 in. H_2_0 (7.5 Pa) of positive pressure to the outside environment. The room was equipped with typical OR air flow obstructions including surgical lights, tables and medical equipment. Mixing was performed in a plastic bowl (HDPE) with an 11.4 cm opening. The bowl was placed on a table in the perimeter of the room. A calibrated Aeroqual Series 500 portable volatile organic compound (VOC) sensor (Gas Sensing, Inwood, IA) was used to measure VOCs produced during bone cement mixing. This sensor utilizes gas sensitive semiconductor technology and measures from 0 to 500 ppm with a resolution of 1 ppm and a response time of 30 s. The accuracy of the factory calibration is <  ± 5 ppm + 10% and the temperature and relative humidity range of the sensor are 0–40 °C and 10–90%, respectively. The sensor collected 1 data point per minute. The sensor was placed approximately 53 cm above the bottom of the mixing bowl and approximately 1.52 m (five ft.) above the floor of the OR. The sensor inlet was aimed downwards toward the bowl. A second sensor was placed at the opposite wall of the OR approximately 4.6 m away from the first sensor, however, this sensor showed negligible changes. The powdered component containing PMMA/PMA and polymerization initiator was added first. The liquid component was added to the powder. After adding the liquid component, the mixture was manually stirred with a wooden spatula for 5 min. The material increased in viscosity but did not become rigid until after the mixing process. VOC concentration in ppm was recorded electronically by the sensor every minute. The background levels of VOC in the OR prior to mixing ranged from 0 to 1 ppm. An Aerocure Vac (Aerobiotix, Miamisburg, OH) PNP unit was used to remove VOC from the mixing site. The device was equipped with a 1.3 cm carbon filter for adsorbing VOCs. The device also contains a HEPA filter and UVC lamps for air disinfection. The inlet tube was covered with a sterile drape that allowed air to flow into the device without compromising the airflow cubic ft. per minute (cfm). Two positions of the funnel were tested (See Fig. 2). In the first position the funnel was placed 8 cm away from the top of the bowl and 20 cm higher than the top of the bowl. In the second position the funnel was placed 36 cm away from the top of the bowl and 36 cm higher than the top of the bowl. Data was collected electronically and downloaded to an external computer for analysis and processing using Aeroqual Series S500 Monitor Software V6.5, Microsoft Excel and OriginPro 2022b. A Mann–Whitney test was used to compare the VOC concentrations from five measurements between the control and PNP cases. The tests were considered significant at p < 0.05.

## Results and discussion

**Figure 3:**
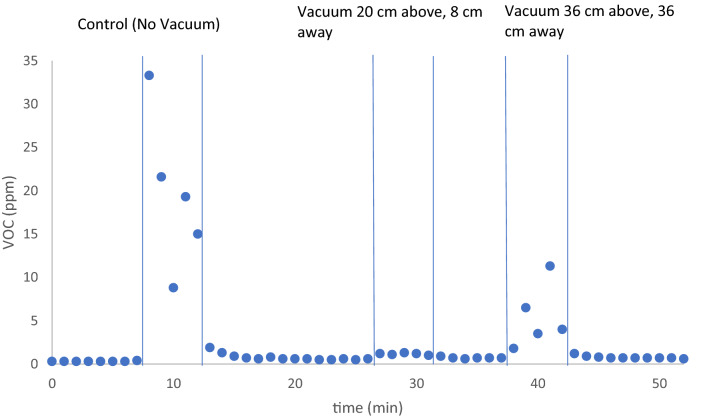
Detected VOC levels before during and after mixing are shown for the control (no vacuum) and for two positions of the vacuum nozzle, the first being 20 cm above and 8 cm away from the bowl, and the second being 36 cm above and away from the bowl. A fresh batch of bone cement was mixed for each condition. The vertical lines indicate the start and end of mixing.

Data for the three tested conditions (control, 20 cm and 36 cm) are shown in Fig. 3. Descriptive statistics of the data are presented in Table 1. A control experiment in which VOC levels were measured during mixing of the two-component bone cement formulation without the presence of vacuum was performed. Upon mixing the powder and liquid at room temperature, the mixture gradually increased in viscosity over the course of the five-minute procedure, becoming more difficult to stir as the polymerization reaction proceeded. The material did not become rigid until several minutes after mixing was complete. The local sensor showed large increases in VOC levels relative to the initial background level of 0.4 ppm as shown in Fig. 3. The vertical lines in the graph indicate the start and end point of mixing. The maximum VOC increase recorded relative to the background was 33 ppm. The average VOC concentration within the five-minute mixing period was 19 ppm with a standard deviation of 9 ppm. The median value was also 19 ppm. The sensor stationed on the opposite side of the OR showed negligible changes in VOC concentration. These results show that detectable and appreciable levels of VOCs are present in the local environment where mixing occurs.

**Table 1:** Average and median VOC values for PNP distances of 20 and 36 cm.

Distance (cm)	Average (ppm)	Median (ppm)	Range (ppm)
20	0.6 (0.1)	0.6 (0.5, 0.6)	0.4–0.7
36	5 (4)	3 (3, 6)	1.1–10.6
Control (No PNP)	19 (9)	19 (15, 21)	8.4–32.9

Averages and standard deviations are written as average (standard deviation). Medians and interquartile ranges are written as median (Q1, Q3).

In order to remove the bone cement fumes from the local environment and minimize inhalation risks, a PNP unit was positioned near the mixing bowl. The PNP unit employed in this study contains an adjustable nozzle which allows for removal of contaminants at specified point sources. The inlet of the PNP nozzle was placed towards the mixing bowl and angled downward at approximately 45° relative to the top of the bowl. In the first position (Fig. 2), the top of the nozzle was approximately 20 cm above the height of the bowl and the bottom of the nozzle was approximately 8 cm from the side of the bowl. When the vacuum was *on*, bone cement fumes from a freshly mixed batch were barely detectable (Fig. 3), showing that the PNP efficiently removes concentrated fumes from the local environment where the formulation is manually mixed. The maximum observed increase in VOC concentration relative to the background was approximately 0.7 ppm. The average and median values over the five-minute period were both 0.6 ppm and the standard deviation was 0.1 ppm. At this distance, the PNP reduces the average and median rise in VOC concentration by approximately 97% relative to the control (*p* < 0.05). Again, the sensor placed near the opposite wall of the OR showed negligible changes. The PNP effectively prevents concentrated bone cement fumes from rising approximately 53 cm above the bowl when placed 20 cm above and 8 cm away from the edge of the mixing bowl, improving the local air quality in the perimeter of the OR and minimizing inhalation of VOCs by OR staff. As noted above, the sensor is approximately 1.52 m above the floor, which is slightly lower than the average adult height worldwide (1.59 m for females, 1.71 m for men)^10^.

In order to gain insight into the distance dependence of the PNP unit on VOC reduction, the lateral and vertical distance of the vacuum inlet was increased to 36 cm. A fresh batch of bone cement formulation was again mixed and the VOC levels were monitored as before. At this increased distance, the PNP was able to significantly reduce the concentration of VOC levels reaching the sensor. The maximum increase detected was approximately 11 ppm. The average and median values over the 5-min period were 5 and 3 ppm, respectively, and the standard deviation was 4 ppm. At this distance, the PNP reduces the average and median rise in VOC concentration by approximately 75% and 83%, respectively, relative to the control (*p* < 0.05). These results demonstrate a degree of flexibility in placing the PNP unit for generating a considerable reduction in localized VOC levels. Overall, the results at 8 cm and 36 cm compare well with a previous study that showed vacuum mixing systems reduce bone cement fumes by 50–75% compared to open bowl mixing^12^. In an ideal setting the PNP would be placed as close to the opening of the mixing bowl as possible without compromising a healthcare worker’s ability to sufficiently mix the formulation.

## Conclusions

We have shown that a PNP device can be used to minimize exposure to fumes that evolve during bone cement mixing. When the nozzle is 20 cm above the bowl and 8 cm away, the fumes are effectively removed before they are able to reach a sensor stationed 53 cm above the mixing bowl. As expected, increasing the distance between the vacuum nozzle and mixing vessel increases the level of detectable fumes, however, a considerable reduction in VOC levels can still be achieved even at distances of 36 cm.

## Data Availability

All data generated or analysed during this study are included in this published article.

## Abbreviations

PMA: Poly (methyl acrylate)
PMMA: Poly (methyl methacrylate)
PNP: Portable negative pressure
VOC: Volatile organic compound
